# A phase II study of preoperative capecitabine in women with operable hormone receptor positive breast cancer

**DOI:** 10.1002/cam4.164

**Published:** 2014-01-27

**Authors:** Sara M Tolaney, Joon Jeong, Hao Guo, Jane Brock, Daniel Morganstern, Steven E Come, Mehra Golshan, Jennifer Bellon, Eric P Winer, Ian E Krop

**Affiliations:** 1 Department of Medical Oncology, Dana-Farber Cancer InstituteBoston, Massachusetts; 2 Department of Surgery, Gangnam Severance Hospital, Yonsei UniversitySeoul, Korea; 3 Department of Biostatistics and Computation Biology, Dana-Farber Cancer InstituteBoston, Massachusetts; 4 Department of Pathology, Brigham and Women's HospitalBoston, Massachusetts; 5 Department of Medical Oncology, Faulkner HospitalBoston, Massachusetts; 6 Department of Medical Oncology, Beth Israel Deaconess Medical CenterBoston, Massachusetts; 7 Department of Surgery, Brigham and Women's HospitalBoston, Massachusetts; 8 Department of Radiation Oncology, Dana-Farber Cancer Institute and Brigham and Women's HospitalBoston, Massachusetts

**Keywords:** Breast, cancer, capecitabine, chemotherapy, preoperative

## Abstract

Conventional preoperative chemotherapy regimens have only limited efficacy in hormone receptor positive (HR+) breast cancer and new approaches are needed. We hypothesized that capecitabine, which is effective in metastatic breast cancer, may be an active preoperative treatment for HR+ breast cancer. Women with HR+, HER2-negative operable breast cancer received capecitabine, 2000 mg/m^2^ daily in divided doses for 14 days, followed by a 7-day rest period. Treatment was repeated every 21 days for a total of four cycles. The primary endpoint of the study was to determine the rate of pathological complete response (pCR). Because of slow accrual, the study was closed after 24 patients were enrolled. Three patients had a complete clinical response, and eight patients had a partial clinical response, for an overall clinical response rate of 45.8%. There were no cases of pCR. Of the 22 patients who had pathological response assessment by the Miller–Payne grading system, there were six grade 3 responses, and no grade 4 or 5 responses. Toxicity was manageable: the only grade 3 toxicities observed were one case each of diarrhea, palmar plantar erythrodysesthesia, hypokalemia, and mucositis. There was no association between baseline levels, or change in level from baseline to cycle 1, or from baseline to time of surgery, of thymidine phosphorylase (*TYMP*), thymidylate synthase (*TYMS*), dihydropyrimidine dehydrogenase (*DPYD*), or Ki67 and pathological, clinical, or radiographic response. Preoperative capecitabine is a well-tolerated regimen, but appears not lead to pCR when used as monotherapy in HR+ breast cancer.

## Introduction

Hormone receptor positive (HR+) breast cancer (defined as estrogen receptor [ER] and/or progesterone receptor expressing tumors) accounts for 60–70% of breast cancers. While chemotherapy has a role in the treatment of HR+ breast cancer, there is compelling evidence that it is less effective in HR+ tumors than in hormone receptor negative (HR−) tumors, making treatment decisions about the use of chemotherapy complex. A retrospective subset analysis of three consecutive randomized trials conducted by the Cancer and Leukemia Group B (CALGB) found that patients with node-positive HR− breast cancer benefited more from recent improvements in adjuvant chemotherapy than do those with HR+ tumors 1. In that study, the absolute improvement in 5-year disease-free survival (DFS) was 22.8% for HR− patients, as compared with only 7.0% for HR+ patients, and the improvement in overall survival (OS) was 16.7% and 4.0%, respectively. More direct evidence comes from analyses of patients treated with preoperative chemotherapy. The European Cooperative Trial in Operable breast cancer (ECTO) investigated the efficacy of doxorubicin/paclitaxel followed by cyclophosphamide, methotrexate, and fluorouracil (CMF) and found a 12% pathological complete response (pCR) rate in those with HR+ breast cancer compared to a 42% pCR rate in those with HR− breast cancer 2. This observation is further supported by work suggesting that the probability of patients with HR− tumors obtaining a pCR is significantly higher than those with HR+ tumors 3–5. Data from these studies demonstrate that patients with HR+ breast cancers derive only minimal benefit from standard chemotherapy, suggesting other treatment approaches must be explored. Preoperative treatment provides an optimal setting to investigate alternative therapeutic options for HR+ breast cancer.

Capecitabine has significant antitumor activity in metastatic breast cancer, with response rates of ∼25% 6,7. At the time this trial was initiated, there were data from a randomized phase II study comparing single-agent capecitabine to CMF in patients with metastatic breast cancer who were age 55 or older demonstrating the response rate to capecitabine alone was superior to CMF (25% vs. 16%). These data suggested that capecitabine may be an alternative to combination chemotherapy.

Given the limited benefit seen from standard chemotherapy in HR+ breast cancer, identifying less toxic, more effective regimens is of interest. Capecitabine has a favorable safety profile, and its efficacy in the metastatic setting is encouraging, making it an ideal candidate. Capecitabine, a 5-fluorouracil (5-FU) prodrug, was designed to preferentially deliver active 5-FU to tumor cells. This selectivity arises from the differential expression of thymidine phosphorylase (*TYMP*) which converts capecitabine to 5-FU and which has significantly higher activity in cancer cells than in normal tissue 8,9. Because *TYMP* is required to convert capecitabine to 5-FU, it is rational to hypothesize that the clinical response to capecitabine is dependent on intratumoral *TYMP* levels. That is, tumors with higher levels of intratumoral *TYMP* may be more likely to respond to capecitabine 10. Conversely, because thymidylate synthase (*TYMS*) is the molecular target of 5-FU, tumors with higher levels of *TYMS* may be relatively resistant to capecitabine. Similarly, tumors with higher levels of dihydropyrimidine dehydrogenase **(***DPYD*) may also be less responsive to capecitabine because this enzyme is chiefly responsible for 5-FU degradation and thus higher levels of *DPYD* may lead to lower levels of intratumoral 5-FU. The preoperative setting is an ideal situation to not only test efficacy, but also explore biomarkers of response to capecitabine.

## Methods

### Patients

A single-arm, open-label, phase II trial was conducted in women with newly diagnosed, untreated, operable breast cancer at Dana-Farber Cancer Institute, Brigham and Women's Hospital, Faulkner Hospital, and Beth Israel Hospital (all in Boston, MA). Eligible patients were ≥18 years who were diagnosed with operable invasive breast cancer. Patients were required to have a primary tumor ≥1 cm by radiographic imaging and/or palpation. Patients with bilateral cancers were eligible provided that at least one primary tumor met eligibility requirements. Tumors were required to express ER and/or progesterone receptor by immunohistochemistry (tumors with any positive staining were considered eligible), and be HER2-negative. Patients were required to be without evidence of metastatic cancer, and have an ECOG performance status of 0 or 1. Key exclusion criteria included patients with evidence of inflammatory breast cancer, known dihydropyrimidine dehydrogenase (*DPYD*) deficiency, uncontrolled intercurrent illness, and malabsorption syndrome.

### Study treatment

Patients received treatment with oral capecitabine 2000 mg/m^2^ divided into twice daily dosing for 14 consecutive days, followed by a 7-day rest period. Treatment was repeated every 21 days for a total of four cycles (1 cycle = 21 days). The dose of capecitabine was rounded up to the nearest dose that could be delivered with 500 mg tablets. The dose was recalculated based on body surface area prior to the start of each cycle. All patients underwent pretreatment sentinel node evaluation if clinically node negative, or fine-needle aspiration (FNA) of a palpable axillary lymph node, if present. If the FNA of a palpable axillary lymph node was negative, they then underwent sentinel node biopsy. Patients also underwent research core biopsies of the breast mass at baseline, and then again 11–14 days after starting study treatment. A breast MRI was performed at baseline and after completion of study treatment. Patients underwent primary breast surgery, not less than 7 days and not more than 35 days after the last dose of capecitabine. Patients with a positive pretreatment axillary FNA or sentinel lymph node biopsy had a completion axillary dissection at time of definitive surgery. Local therapy of breast-conserving therapy or mastectomy was left to the discretion of the treating breast surgeon. Appropriate postoperative adjuvant chemotherapy, hormonal therapy, and radiation therapy was determined by the patient's treating physician.

Capecitabine doses were reduced or discontinued based on tolerability. Events necessitating dose-reduction included recurrent grade 3 neutropenia, grade 3 neutropenia with grade ≥2 fever, grade 4 thrombocytopenia, grade ≥2 diarrhea, grade ≥2 hand–foot syndrome, CrCl < 50, and any grade ≥2 nonhematological toxicity. If a patient required a dose delay of capecitabine of more than 3 weeks, the patient was withdrawn from the study due to toxicity.

### Predictive markers

Intratumoral levels of *TYMP*,*TYMS*,*DPYD*, and Ki67 were determined at baseline, at the end of the first cycle of therapy, and at the time of surgery. Total RNA was extracted from frozen OCT blocks using the RNeasy® Lipid Tissue Mini Kit according to manufacturer instructions (Qiagen, Germantown, MD). Reverse transcription to complementary DNA (cDNA) was performed using QuantiTect Reverse Transcription Kit (Qiagen).TaqMan primers and probes for *TYMP*,*DPYD*, and *TYMS* genes were ordered from TaqMan® Gene Expression Assays (Hs00157317_m1, Hs00559278_m1, Hs00426586_m1, respectively, Applied Biosystems, Foster City, CA). *β*-glucuronidase was used as an internal control. A total of 50 ng of cDNA was mixed with the TaqMan primers and probes, RNase-free water, and TaqMan Universal PCR Master Mix. Real-time PCR amplification and data analyses were carried out using a StepOnePlus™ Real-Time PCR System (Applied Biosystems) according to the manufacturer's protocol. Each sample was assayed in triplicate in a MicroAmp optical 96-well plate (Life Technologies, Grand Island, NY). The thermocycling condition was 2 min at 50°C and a 10 min incubation at 95°C, followed by 40 two-temperature cycles of 15 sec at 95°C and 1 min at 60°C.

### Statistical analysis

This trial was designed as a two-stage study with a primary objective of determining the pCR rate following four cycles of preoperative capecitabine in women with HR+ and HER2-negative operable breast cancer. It was planned that in the first phase, 42 patients would be entered, and if 0 or 1 pCRs were observed, accrual would terminate. If two or more pCRs were observed, another 38 patients would be entered, for a total of 80 patients. If a total of six or greater pCRs were observed out of 80 patients, the treatment would be declared worthy of further study. There would be a 50% chance of stopping accrual early if the true pCR rate was 4%, a 37% chance of stopping accrual early if the true pCR rate was 5%, and a 7% chance of stopping early if the true pCR rate was 10%. Overall, there was a 10% probability of declaring the study regimen worthy of further study if the true pCR rate was 4% and an 80% probability if the true pCR rate was 10%. The study, however, was terminated early due to slow accrual, with 24 patients enrolled. The 95% confidence intervals of the response rates were calculated using Wilson Score method 11. Toxicity data was reviewed and summarized by grade. Biomarker (*TYMP*,*TYMS*,*DPYD*) assessments at baseline, during cycle 1 (11–14 days after start of therapy), and at surgery were tabulated. Wilcoxon signed rank test was used to test the difference of *TYMP* levelsat baseline and cycle 1.

## Results

The trial opened in September 2004 and closed in September 2007 due to slow accrual. There were 24 patients who enrolled in the trial. Two patients came off study early, and therefore were not evaluable for efficacy. One patient was taken off study after experiencing early-onset grade 3 diarrhea and palmar-plantar erythrodysesthesia (PPE), and another patient decided to withdraw consent during cycle 1 of treatment due to grade 1 nausea. Twenty-two patients went on to complete protocol treatment.

Of the 24 patients enrolled, 14 patients (58%) had evidence of nodal positivity at baseline, either by pretreatment sentinel node procedure or FNA of a palpable lymph node. The median tumor size by MRI imaging at baseline was 3.4 cm, with 83% of patients having tumors that were greater than 2 cm. All patients had HR-positive, HER2-negative tumors, and 75% were histologically grade 2 or higher. (Table 1).

**Table 1:** Patient characteristics.

	Number of patients (Total *N* = 24)
Age (years)	
Median (range)	53 (29–71)
Ethnicity	
Caucasian	23 (95.8%)
Hispanic	1 (4.2%)
Clinical tumor size	
T1	4 (16.7%)
T2	14 (58.3%)
T3	6 (25.0%)
T4	0 (0%)
Tumor size (cm) based on baseline MRI	
Median (range)	3.4 (1.0–9.0)
≤2	4 (16.7%)
2–5	15 (62.5%)
>5	5 (20.8%)
Clinical nodal status	
Negative	13 (54.2%)
Positive	11 (45.8%)
Pathological nodal status	
Positive	14 (58.3%)
Performed FNA only	6 (25.0%)
Performed sentinel node biopsy only	6 (25.0%)
Performed both FNA and sentinel node biopsy	2 (8.3%)
Negative	10 (41.6%)
Performed sentinel node biopsy only	8 (33.3%)
Performed both FNA and sentinel node biopsy	2 (8.3%)
Receptor status	
ER positive	24 (100%)
PR positive	24 (100%)
HER2-negative	24 (100%)
Ki67 expression	
<5	7 (29.2%)
≥5	17 (70.8%)
Grade	
1	6 (25%)
2	12 (50%)
3	6 (25%)

Treatment was generally well tolerated, with the most common toxicities being PPE, diarrhea, anemia, and nausea. There were only four grade 3 events (PPE, diarrhea, hypokalemia, and mucositis) with no grade 4 events. (Table 2).

**Table 2:** Toxicity of preoperative capecitabine therapy.

Toxicity	Grade 1–2, *n* (%)	Grade 3, *n* (%)	Grades 1–3,%
Palmar-plantar erythrodysesthesia	19 (79.2%)	1 (4.2%)	83.3%
Diarrhea	15 (62.5%)	1 (4.2%)	66.7%
Anemia	9 (37.5%)	0 (0%)	37.5%
Nausea	9 (37.5%)	0 (0%)	37.5%
Fatigue	8 (33.3%)	0 (0%)	33.3%
Mucositis	4 (16.7%)	1 (4.2%)	20.8%
AST/ALT	4 (16.7%)	0 (0%)	16.7%
Abdominal pain	4 (16.7%)	0 (0%)	16.7%
Anorexia	3 (12.5%)	0 (0%)	12.5%
Elevated bilirubin	3 (12.5%)	0 (0%)	12.5%
Hyperglycemia	3 (12.5%)	0 (0%)	12.5%
Insomnia	3 (12.5%)	0 (0%)	12.5%
Leukopenia	3(12.5%)	0 (0%)	12.5%
Rash	3 (12.5%)	0 (0%)	12.5%
Hypokalemia	0 (0%)	1 (4.2%)	4.2%

There were no pCRs among the 24 patients (pCR rate 0%, 95% CI: 0–13.8). All cases were centrally reviewed for Miller–Payne response 12. This grading system is used to assess pathological response of the tumor in the breast after preoperative chemotherapy. A grade 1 response indicates there was no change in the overall cellularity of the tumor; a grade 2 response indicates there was up to a 30% loss in overall cellularity; a grade 3 response indicates a 30–90% reduction in tumor cells; a grade 4 response indicates a marked disappearance of tumor cells such that only small clusters of widely dispersed individual cells remain (more than a 90% loss of tumor cells); and a grade 5 response indicates no malignant tumor cells are identifiable; ductal carcinoma in-situ may be present. This system does not include the assessment of axillary nodes. In this study, all patients had a Miller–Payne 3 or lower response. (Table 3).

**Table 3:** Response data to preoperative capecitabine therapy.

Pathological response	Number of patients (%)	Pathological complete response rate (95% CI)
pCR	0 (0)	0 (0–13.8)
Not pCR	22 (91.7)	
Unevaluable^1^	2 (8.3)	
Best overall response	Number of patients (%)	Objective response rate (95% CI)
Radiographic		
CR	0 (0)	37.5 (21.2–57.3)
PR	9 (37.5)	
SD	11 (45.8)	
PD	2 (8.3)	
Unevaluable^1^	2 (8.3)	
Clinical		
CR	3 (12.5)	45.8 (27.9–64.9)
PR	8 (33.3)	
SD	11 (45.8)	
PD	0 (0)	
Unevaluable^1^	2 (8.3)	
Miller–Payne		
1	10 (41.7)	
2	6 (25.0)	
3	6 (25.0)	
4	0 (0)	
5	0 (0)	
Unevaluable^1^	2 (8.3)	

pCR, pathological complete response; CR, complete response; PR, partial response; SD, stable disease; PD, progressive disease.

1 Two patients were off treatment after Cycle 1.

Clinical responses were assessed after completion of four cycles of capecitabine and prior to surgery, and there were three cases of complete clinical response, with eight cases of partial clinical response, consistent with a 46% clinical objective response rate. There were no cases of clinical progression while on study. (Table 3).

Radiographic response was assessed by MRI imaging by comparing tumor measurements at baseline with those obtained prior to surgery. A complete response (CR) was defined as complete disappearance of tumor, a partial response (PR) was defined as a greater than or equal to 30% decrease in longest diameter, progressive disease (PD) was defined as a greater than or equal to 20% increase in longest diameter, and stable disease (SD) was defined as tumor measurements not qualifying as a CR, PR, or as PD. Overall, there was a 37.5% radiographic response rate, with no cases of CR. (Table 3).

Levels of *TYMP*,*TYMS*,*DPYD*, and Ki67 were assessed at baseline, during cycle 1 (11–14 days after start of therapy), and at time of surgery (Table 4). *TYMP* levels were significantly higher during cycle 1(11–14 days after start of therapy) than at baseline (*P* = 0.004). There was no significant association between baseline biomarker levels or change in biomarker level (baseline to cycle 1 or baseline to surgery) and clinical, radiographic, or Miller–Payne response.

**Table 4:** Biomarkers on tissue collected at baseline, after one cycle, and at time of surgery.

Biomarker	Baseline (fold expression)	Cycle 1 (fold expression)	Surgery (fold expression)
Thymidine phosphorylase (*TYMP*)			
*N*	21	17	13
Median (range)	0.58 (0.11–1.68)	1.36 (0.41–10.79)	0.39 (0.09–1.66)
Thymidylate synthase (*TYMS*)			
*N*	21	17	13
Median (range)	0.22 (0.07–0.63)	0.32 (0.07–1.1)	0.16 (0.08–0.77)
Dihydropyrimidine dehydrogenase (*DPYD*)			
*N*	21	16	13
Median (range)	0.42 (0.11–1.76)	0.67 (0.24–1.94)	0.30 (0.15–1.19)

## Discussion

Numerous trials have investigated the use of preoperative chemotherapy with the goal of improving breast-conserving therapy rates. The National Surgical Adjuvant Breast and Bowel Project B-18 trial demonstrated that doxorubicin and cyclophosphamide administered as neoadjuvant or adjuvant therapy had equivalent outcomes in terms of DFS and OS 13. Additionally, neoadjuvant chemotherapy has been shown to increase the rate of breast-conserving surgery (BCS) without adversely affecting DFS or OS 14. However, preoperative chemotherapy produces lower rates of pathological complete responses in HR+ breast cancers compared to HR− breast cancers 5. Moreover, conventional combination chemotherapy may be associated with substantial toxicity. Therefore, there is a great need for effective alternatives to anthracycline and taxane-based chemotherapy for HR+ breast cancer.

Capecitabine was postulated to be an attractive alternative to standard preoperative chemotherapy as it is generally well tolerated, and in the metastatic setting was found to have response rates similar to CMF 6,7. This study is the first to evaluate preoperative capecitabine given as monotherapy for HR+ breast cancer. Data that emerged after this trial completed accrual demonstrated that standard adjuvant combination chemotherapy is superior to capecitabine in patients with early-stage breast cancer who are ≥65 years of age. An unplanned subgroup analysis demonstrated that the major benefit of standard chemotherapy occurred in patients with HR negative breast cancer 15. Additionally, preoperative trials have failed to demonstrate a benefit from the addition of capecitabine to standard chemotherapy 16,17. This study supports these findings, as preoperative capecitabine was unable to achieve pCRs in HR+ breast cancer.

Given the small numbers of patients, and the overall limited efficacy of the capecitabine regimen, it is difficult to determine association of *TYMP*,*TYMS*, and *DPYD* with response. Higher levels of *TYMP* have been associated with higher rates of pathological response to preoperative capecitabine or 5-FU in colorectal cancer 18,19, and to prolonged time to progression from capecitabine in metastatic breast cancer 20,21, but in this study, there did not appear to be a trend with baseline *TYMP* and clinical, radiographic, or Miller–Payne response. However, as noted, it is difficult to draw conclusions with the small numbers of patients in each response group.

Interestingly, there was a significant increase in *TYMP* from baseline to the biopsy performed at cycle 1, but levels decreased in the surgical specimen. This may suggest early induction of *TYMP* with initiation of capecitabine therapy. *TYMP* has been shown to increase in tumor samples of patients receiving anthracycline-and taxane-based preoperative chemotherapy for breast cancer and this observation provided the rationale for several studies which evaluated the addition of capecitabine to taxane-based therapy 22–24. *TYMP* expression has been significantly associated with expression of TNF-α, IL-1a, and monocyte chemoattractant protein (MCP)-1 25. It is possible that increases in *TYMP* may be triggered by increases in concentrations of these immune mediators, which may be stimulated by the initiation of chemotherapy.

Our results suggest that capecitabine monotherapy appears to have limited efficacy HR+HER2-breast cancer, consistent with other studies which have failed to identify a role for this agent in the treatment of early-stage breast cancer. Other approaches will need to be explored to improve preoperative response rates in HR+, HER2-breast cancer.
